# Single‐cell atlas of healthy vocal folds and cellular function in the endothelial‐to‐mesenchymal transition

**DOI:** 10.1111/cpr.13723

**Published:** 2024-09-08

**Authors:** Danling Liu, Yunzhong Zhang, Luo Guo, Rui Fang, Jin Guo, Peifang Li, Tingting Qian, Wen Li, Liping Zhao, Xiaoning Luo, Siyi Zhang, Jun Shao, Shan Sun

**Affiliations:** ^1^ Department of Otorhinolaryngology, Guangdong Provincial People's Hospital (Guangdong Academy of Medical Sciences), Guangdong Cardiovascular Institute Southern Medical University Guangzhou China; ^2^ ENT Institute and Otorhinolaryngology, Innovation Center, Affiliated Eye and ENT Hospital, Key Laboratory of Hearing Medicine of NHFPC, State Key Laboratory of Medical Neurobiology Fudan University Shanghai China; ^3^ Key Laboratory of Optoelectronic Devices and Systems of Ministry of Education and Guangdong Province, Institute of Microscale Optoelectronics and Otolaryngology Department and Biobank of the First Affiliated Hospital, Shenzhen Second People's Hospital, Health Science Center Shenzhen University Shenzhen China

## Abstract

The vocal fold is an architecturally complex organ comprising a heterogeneous mixture of various layers of individual epithelial and mesenchymal cell lineages. Here we performed single‐cell RNA sequencing profiling of 5836 cells from the vocal folds of adult Sprague–Dawley rats. Combined with immunostaining, we generated a spatial and transcriptional map of the vocal fold cells and characterized the subpopulations of epithelial cells, mesenchymal cells, endothelial cells, and immune cells. We also identified a novel epithelial‐to‐mesenchymal transition‐associated epithelial cell subset that was mainly found in the basal epithelial layers. We further confirmed that this subset acts as intermediate cells with similar genetic features to epithelial‐to‐mesenchymal transition in head and neck squamous cell carcinoma. Finally, we present the complex intracellular communication network involved homeostasis using CellChat analysis. These studies define the cellular and molecular framework of the biology and pathology of the VF mucosa and reveal the functional importance of developmental pathways in pathological states in cancer.

## Graphical abstract

Dimensionality reduction and clustering analysis of sc‐RNA seq data from healthy SD rats' vocal folds identified five cell populations. Notably, a Krt15+/Col3a1+ cell type with epithelial‐mesenchymal features may play a crucial role in head and neck squamous cell carcinoma.

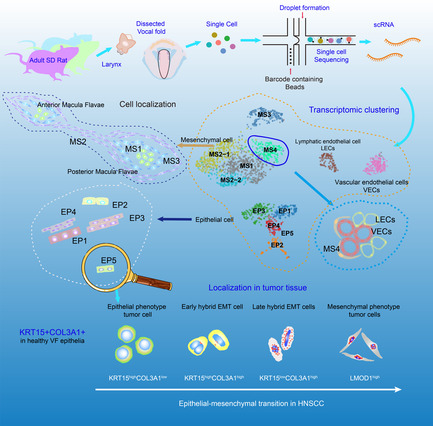

## 1 INTRODUCTION

The vocal folds (VFs) are delicate layered structures serving as the junction of the digestive and respiratory tracts and performing multiple functions such as breathing, phonation, and pathogen defence.^1^ Human VFs, including the outer epithelial layer, the lamina propria layer, and the deepest muscles, are vulnerable to multiple injury factors and environmental insults and therefore suffer a variety of pathologies, the aetiologies of which remain to be fully determined.^2^, ^3^ However, previous studies based on morphological classification and bulk‐RNA sequencing may mask the properties and complexity across cell groups and within the same cell type, especially for less abundant cell types.^4^, ^5^

Single‐cell RNA sequencing (scRNA‐seq) has emerged as a revolutionary technology for identifying new cell types, analysing cellular heterogeneity, and capturing the dynamics of genetic regulation.^5^, ^6^ With the goal of deciphering the cellular composition and the complex cellular interactions in VFs, here we have constructed for the first time an unbiased, systematic scRNA‐seq atlas of VF tissues by profiling 5836 high quality cell transcriptomes from healthy male Sprague–Dawley (SD) rats. Across five major VF cell types, we identified transcriptionally distinct subpopulations and located cell distributions. Notably, to our knowledge, we are the first to discover a new subset of epithelial cells showing both epithelial and mesenchymal features. We therefore hypothesized that this cell type is critical for the epithelial‐to‐mesenchymal transition (EMT), and we confirmed similar genetic specific features in samples from head and neck squamous cell carcinoma (HNSCC) patients. Our findings will therefore serve as a valuable resource for future research seeking to unveil the mechanisms in the physiology and pathophysiology of the VF.

## 2 RESULTS

### 2.1 A cell molecular atlas of healthy VF tissues

**FIGURE 1:**
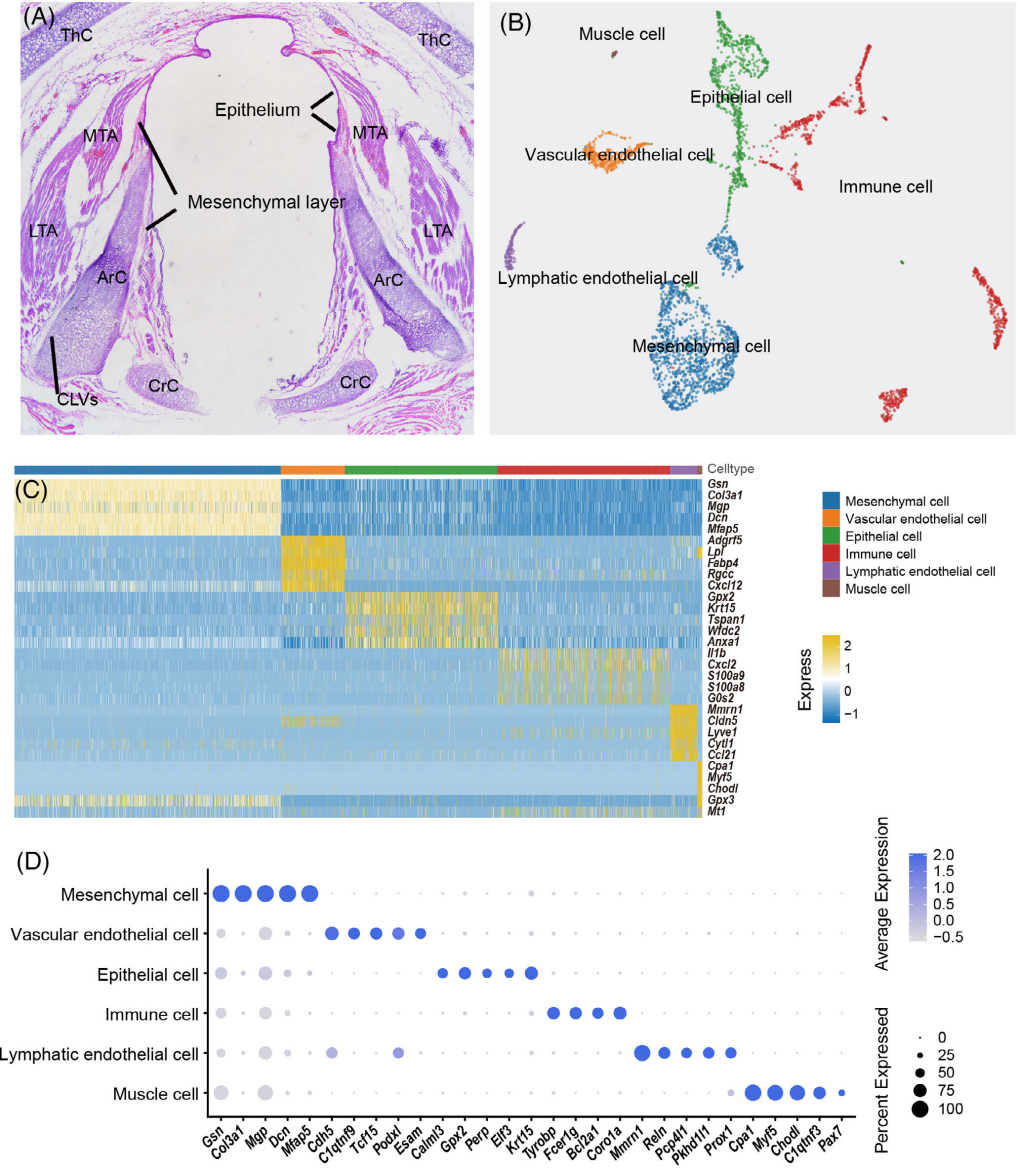
Cellular classification by scRNA‐seq in healthy rat vocal fold (VF) tissue. (A) HE staining of the rat VF in the coronal position. ArC, arytenoid cartilage; CrC, cricoid cartilage; LTA, lateral thyroarytenoid muscle; MTA, medial thyroarytenoid muscle. (B) Uniform Manifold Approximation and Projection showing all separate cell clusters for the VF mucosa, including epithelial cells (green), mesenchymal cells (blue), lymphatic ECs (yellow), vascular ECs (purple), immune cells (red) and muscle cells (brown). (C) Heatmap showing genes (rows) that are differentially expressed across five main cell populations (columns) clustered according to gene expression patterns. (D) DotPlot of example genes identified in each cell cluster. The size of the dot indicates the percentage of cells within a cell type, and the colour indicates the average expression level.

The VF atlas included the epithelial layer, mesenchymal layer, muscles and cartilage (Figure 1A). A total of 5836 high‐quality cells were identified from the VF cell suspension and were subjected to droplet‐mediated scRNA‐seq (10× Genomics Chromium). Five cell populations were next separated using Seurat v4 and were visualized by Uniform Manifold Approximation and Projection (UMAP) analysis. Manual cell annotation was performed for the five cell populations using pre‐established cell type‐specific marker genes, including *Krt15* and *Gpx2‐*marked epithelial cells (EPs, 38.7%), *Cdh5‐*marked endothelial cells (ECs, 25.1%), *Gsn* and *Col3a1‐*marked mesenchymal cells (MSs, 13.2%), immune cells (0.8%), and a few *Pax7* and *Myf5‐*marked muscle cells^7^, ^8^, ^9^, ^10^, ^11^, ^12^, ^13^, ^14^, ^15^ (Figure 1B–D).

To further clarify the cellular heterogeneity, cell populations were analysed with higher resolution based on the gene expression patterns and the distribution of the top marker genes.

### 2.2 Heterogeneity and distribution of VF EPs

**FIGURE 2:**
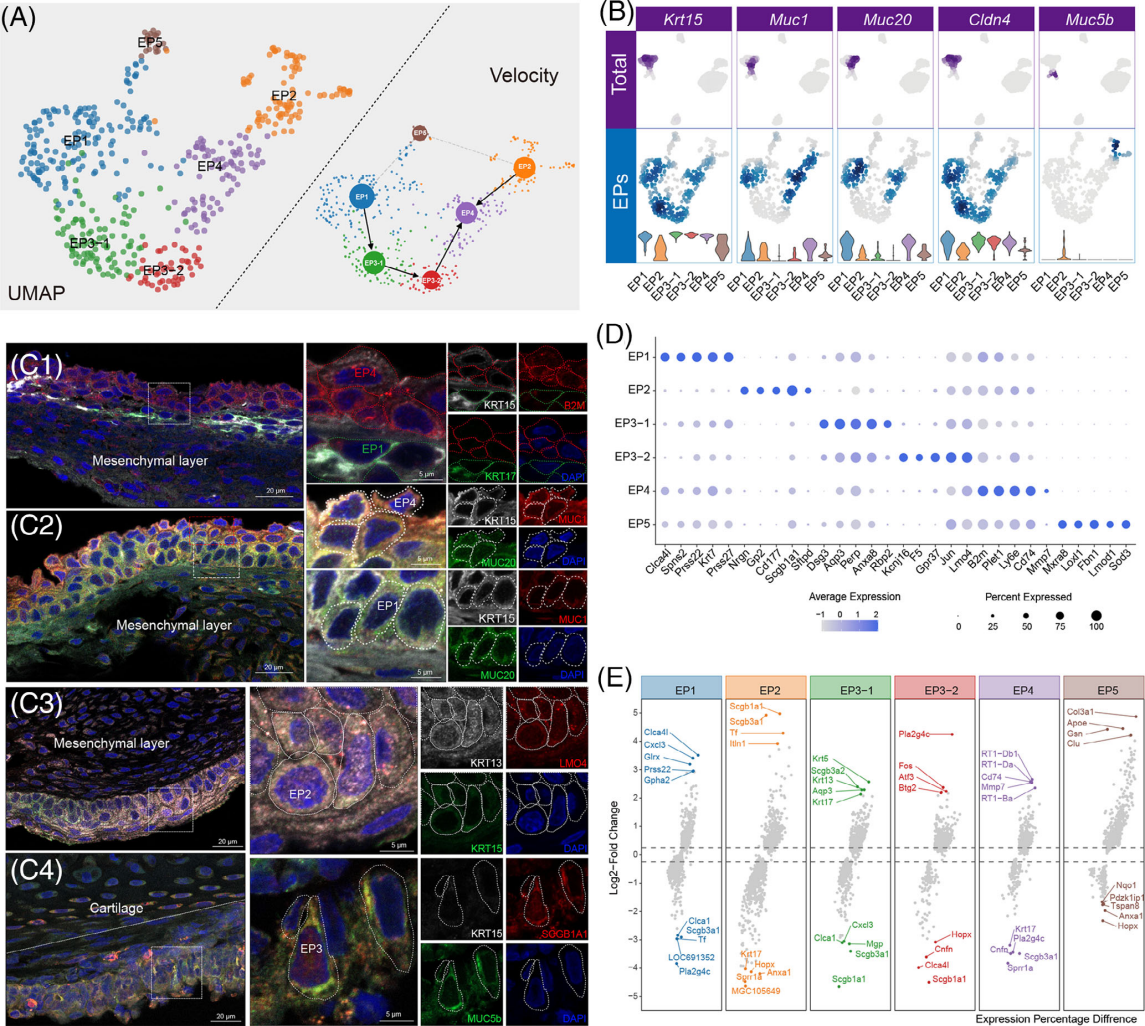
Heterogeneity and distribution of vocal fold (VF) epithelial cells (EPs). (A) Uniform Manifold Approximation and Projection clustering (left) and velocity plot (right) of VF EPs. (B) Violin plot of mucin gene expression and the distribution in VF EPs. (C1) Immunofluorescent staining for KRT15, KRT17 and B2M showing EP1 (basal cells) and EP4 (late suprabasal cells). Red indicates MUC1, green indicates KRT17 and white indicates KRT15. (C2) Immunofluorescent staining for KRT15, MUC1 and MUC20 showing mucin expression in EP1 and EP4. Red indicates B2M, green indicates KRT17 and white indicates KRT15. (C3) Immunofluorescent staining for KRT15, LMO4 and KRT13 showing EP3 (early and mid‐suprabasal cells). Red indicates LMO4, green indicates KRT15 and white indicates KRT13. (C4) Immunofluorescent staining for KRT15, MUC5b and SCGB1A1 showing EP2 (club cells). Red indicates SCGB1A1, green indicates MUC5b and white indicates KRT15. (D) Dot plot of the top gene markers expressed by EPs. The size and colour are illustrated in the same way as in Figure 1. (E) Scatter plot for the distribution of the top gene markers (upper half) and lowly expressed markers (lower half) in EPs.

We identified four regular subpopulations and an unreported subpopulation (EP5) of EP cells (Figure 2A,B). These EP subclusters, from EP1 to EP3‐1, to EP3‐2 and to EP4, constituted a continuum of differentiated stratified squamous EP layers based on velocity analysis, and these were later localized by marker proteins using immunofluorescence staining (Figure 2A,C1–C4). Cluster EP1 was identified as the basal cells expressing both *Krt17* and *Prss22* (Figure 2C1,D,E), which characterized airway epithelial and stratified oesophageal epithelia markers, respectively.^16^, ^17^ EP1 also strongly expressed *Clca4l*, which is highly expressed in the rat olfactory epithelium and in the central nervous system^18^, ^19^ (Figure 2E). EP3 was located in the middle epithelial layer, consisting of the early‐differentiated *Krt13* + *Krt5* + EP3‐1 and the late‐differentiated *Lm*o4+ EP3‐2 with proliferation potential (Figure 2C3,D,E).^20^, ^21^, ^22^ The *B2m* + cluster EP4 was identified as the superficial EPs with highly expressed immune‐related markers, such as *Ly6e*, *Cd74* and RT1 class II proteins, which might be correlated with defensive barrier functions that have not yet been fully clarified (Figure 2C1,D,E).^17^, ^23^, ^24^ In particular, EP2 was identified as club cells or Clara cells which belongs to EPs, with high *Scgb1a1*, *Scgb3a1* and *Tf* expression but lower keratin expression, and these cells are commonly seen in the airways participating in mucus formation and airway defence (Figure 2C4,D,E).^25^, ^26^

We next measured mucin expression among the EP subpopulations, which is involve in the barrier defence of mucosal epithelial surfaces. MUC1 and MUC20 were mainly identified in EP1 and EP4 and were less expressed in EP3 (Figure 2B–C2). Also, EP2 showed distinctive MUC5b expression in the cytoplasm (Figure 2B–C4). Unsupervised gene set variation analysis (GSVA), Gene Ontology (GO) and Kyoto Encyclopedia of Genes and Genomes (KEGG) enrichment revealed the functional abundance and the significant differences among EP subtypes, indicating an increase in intercellular heterogeneity along with differentiation (Figures S1–S3).

**FIGURE 3:**
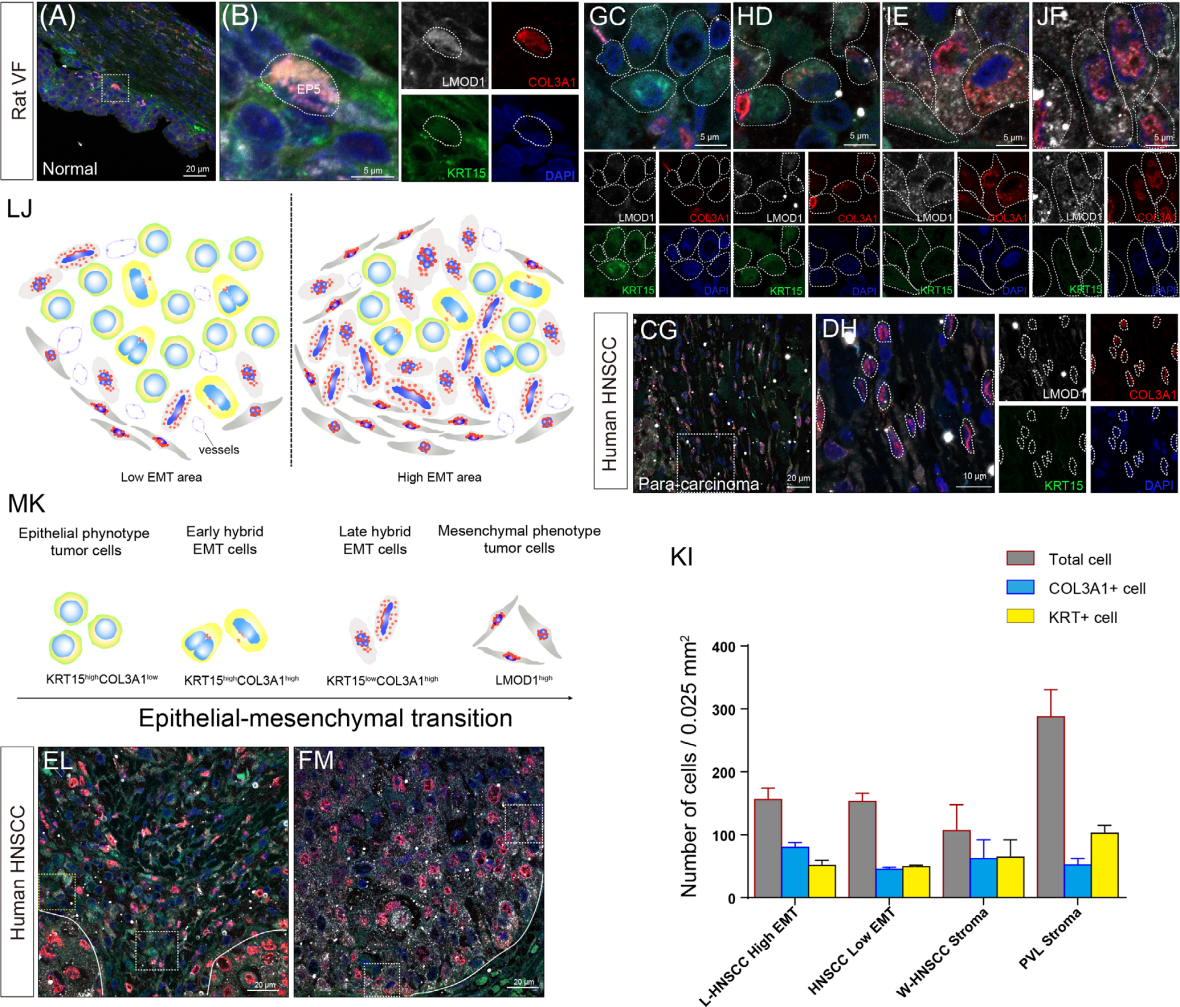
Identification and localization of EP5 cells and epithelial‐to‐mesenchymal transition (EMT) cells. (A, B) EP5 cells at the bottom of the vocal fold epithelia among the EP1 cells, which are morphologically similar. (C–F) Four states of EMT cells, including KRT15^high^COL3A1^low^ cells with a round shape, elongated KRT15^high^COL3A1^high^ cells with enlarged or mitotic nuclei, KRT15^low^COL3A1^high^ cells with higher mesenchymal marker expression and narrow nuclei, and LMOD1^high^ cells with a mesenchymal phenotype with higher mesenchymal marker expression. (G, H) Mesenchymal cells in the para‐cancerous area with flatter and smaller spindle‐like shapes; (I) The ratio of KRT15^high^ and COL3A1^high^ EMT cells in the High EMT area in L‐HNSCC, the Low EMT area in L‐HNSCC, the W‐HNSCC stroma and the proliferative verrucous leukoplakia stroma. (J) A schematic diagram depicting the levels of EMT in the Low EMT area and High EMT area. (K) A schematic diagram of the EMT process from the epithelial phenotype to the hybrid EMT state and finally to the mesenchymal phenotype. (L) The Low EMT area (tumour stroma) showing a higher ratio of KRT15+ EMT cells with erythrocyte infiltration; (M) The High EMT area (tumour parenchyma) showing a higher ratio of COL3A1+ EMT cells.

EP5 was shown to be a unique subtype co‐expressing the epithelial and mesenchymal markers *Krt15* and *Col3a1*. Additionally, EP5 presented multiple extracellular matrix (ECM) markers such as *Fbn1* and *Lxol1*, which are involved in ECM synthesis and remodelling.^27^, ^28^ EP5 also expressed *Lmod1*, which is preferentially expressed in differentiated smooth muscle cells and may regulate sarcomere assembly and cell movements (Figure 2D,E).^29^, ^30^ Although EP5 showed a considerable degree of functional similarity to EP1 (Figure S1), we believed that EP5 might not be a major source for epithelial differentiation because its developmental trajectory is detached from other EPs (Figure 2A), thus making its role in normal VF epithelia more of a mystery. EP5 was marked by COL3A1, KRT15 and LMOD1 and was found scattered among the EP1 basal cells and was morphologically similar but with smaller, flatter nuclei (Figure 3A,B).

The intermediate cell states are more commonly identified in the EMT/mesenchymal‐epithelial transition (MET) process during tumorigenesis and in tissue development, injury repair, and so forth^31^, ^32^ and are usually referred to as partial or hybrid EMT states in malignant tumours.^33^ We therefore investigated the EMT cells in tissues from poorly‐differentiated head and neck squamous cell carcinoma (HNSCC) based on the transcriptomic features of EP5. We identified four EMT cell states as KRT15^high^COL3A1^low^, KRT15^high^COL3A1^high^, KRT15^low^COL3A1^high^ and LMOD1^high^ according to the expression features as well as cellular morphological change. With the increase of COL3A1 and LMOD1 and the decrease of KRT15, the cells became elongated from their round shapes and took on long spindle‐like shapes. This process was accompanied with nuclear hypertrophy, increased mitosis, heterokaryosis and the abnormal accumulation of type III collagen around the nucleus, thus representing an EMT‐like transition (Figure 3C–F). In addition, the mesenchymal phenotype of LMOD1^high^ COL3A1^high^ EMT cells showed more hypertrophic shapes compared to the mesenchymal cells in the para‐cancerous area (Figure 3B,G,H), thus reflecting the complexity of tumour mesenchymal composition. We later determined the EMT area as ‘Low’ and ‘High’ according to the ratio and the distribution of different EMT cells (Figure 3I–K). Of note, all four EMT cell states could be detected in both High and Low EMT areas (Figure 3L,M). However, a higher ratio of the KRT15^low^COL3A1^high^ cells and LMOD1^high^ cells was observed in the High EMT areas, most of which were in the parenchyma. In addition, there were more KRT15^high^COL3A1^low^ and KRT15^high^COL3A1^high^ cells in the Low EMT areas, which were identified at the margin of the parenchyma or in the tumour stroma (Figure 3I–K). Interestingly, more vascular‐like structures were surrounding rather than being centred in the High EMT area (Figure 3L,M). We also detected the EP clusters in tissue from well‐differentiated HNSCC and proliferative verrucous leukoplakia (PVL). We found that the stratified structure of the epithelium was maintained in both tissues despite the obvious epithelial anisotropy accompanied by an increase in COL3A1 and LMOD1. In addition, there was no significant difference (*p* >0.05) between the ratio of KRT15^high^ cells and COL3A1^high^ cells in the well‐differentiated HNSCC, while a higher proportion of KRT15^high^ cells was detected in the PVL tissue (Figure 3I). Thus, gene expression features of EP5 cells are potentially significant for head and neck tumorigenesis.

### 2.3 Endothelial cells of the VF

**FIGURE 4:**
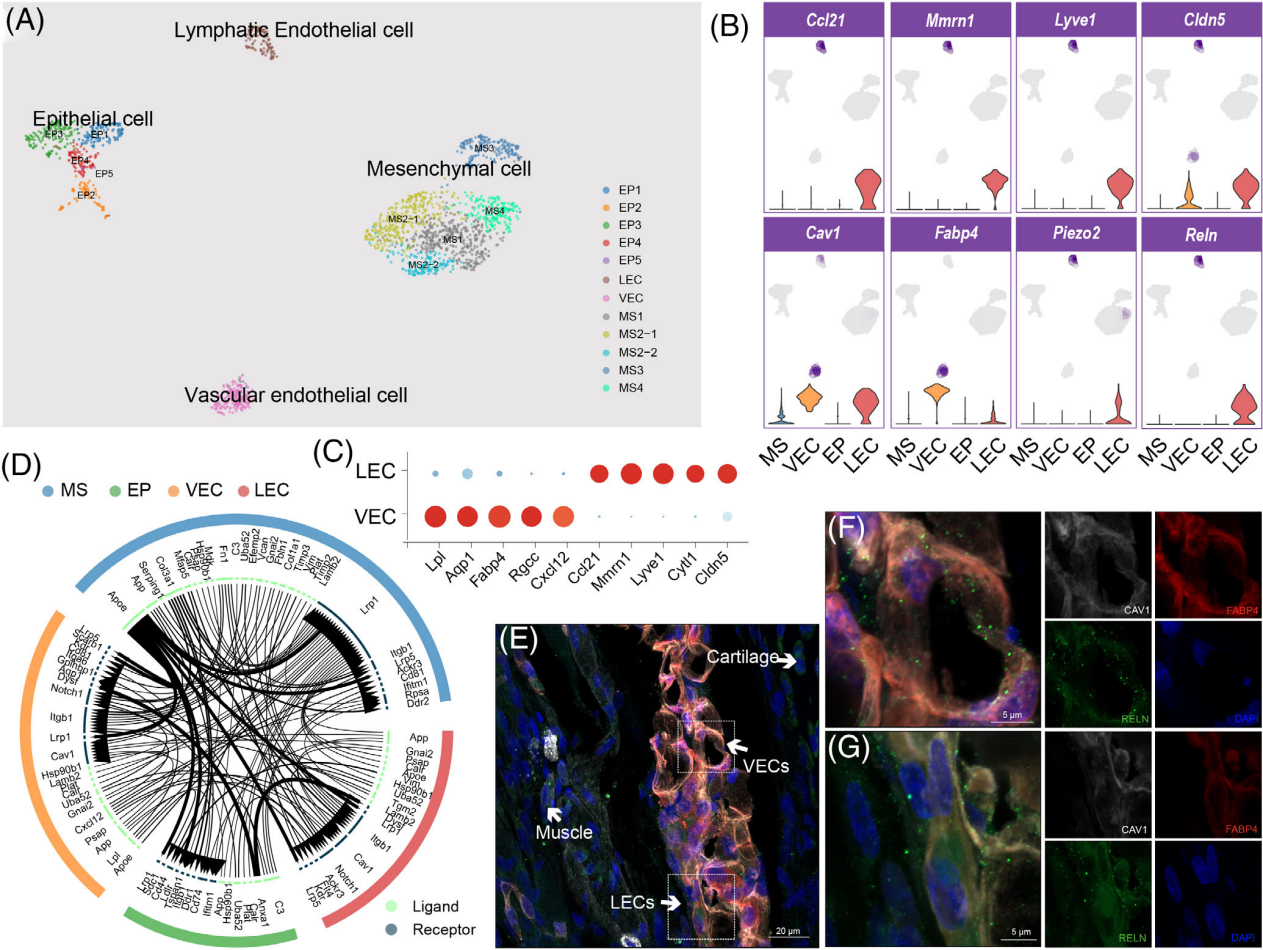
Cell types of the vocal fold (VF) endothelial cells. (A) Uniform Manifold Approximation and Projection of vascular endothelial cells (VECs) and lymphatic endothelial cells (LECs). (B) Violin plot of the top gene markers in VECs and LECs. (C) Dot plot of the gene features expressed by LECs and VECs. The size of the dot indicates the percentage of cells within a cell type, and the red colour represents a high expression level and the blue colour represents a low expression level. (D) Chord plot of 39 cell–cell signalling pathways in VF tissue. (E–G) VECs and LECs form blood vessels and lymphatic vessels, respectively, within muscles and cartilage.

Labelled by the endothelial markers *Cdh5* and *Podxl*,^34^ the ECs could be further divided into lymphatic ECs (LECs) and vascular ECs (VECs) by further gene expression pattern analysis (Figure 4A). The VECs expressed the vascular markers *Esam*, *Fabp4* and *Aqp1*, while the LECs distinctly expressed the lymphatic markers *Reln*, *Lyve1*, *Mmrn1* and *Ccl21* ^9^, ^35^ (Figure 4B,C). Through ligand‐receptor analysis, we observed strong interactions between MSs and VECs and MSs and LECs, respectively (Figure 4D). However, fewer interactions between VECs and LECs were identified. The VECs and LECs were spatially identified by FABP4 and RELN staining mainly located in the anterior commissure and in the interstitial spaces of cartilage and muscles (Figure 4E–G).

### 2.4 MS heterogeneity reflects distinct ECM profiles

**FIGURE 5:**
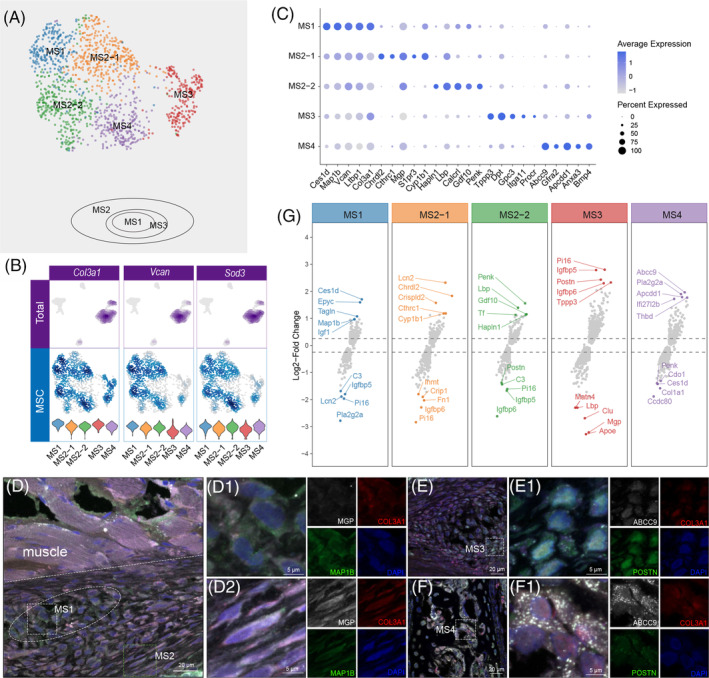
Profiles and spatial location of vocal fold (VF) mesenchymal cells (MSs). (A) Uniform Manifold Approximation and Projection of VF MSs (upper) and a schematic diagram of the distribution of MS1–3. (B) Violin plots of the overlapping gene markers in MS cells. (C) Dot plot of the top gene markers expressed by MSs. The size and colour are illustrated in the same way as in Figure 1. (D–F) Mapping of MS subpopulations. (D1) The MS1 cells with irregular shape in the centre of the ring‐like structure. (D2) The MS2 cells in the outer layer of the rings. (E, E1) The MS3 cells in the middle circle of the rings. (F, F1). The pericyte MS4 cells together with the ECs form the blood vessels in the anterior commissure of the VF. (G) Scatter plot of the expression intensity of the top gene markers in MS cells (upper half) and lowly expressed markers in MSs (lower half).

Four MS subclusters were separated by high‐resolution sub‐clustering analysis (Figure 5A) and were identified as fibroblasts (MS1–3) and pericytes (MS4) according to their top marker genes. The VF MSs generally expressed the MS markers *Vcan* and *Sod3* with tissue‐specific *Col3a1*, which is one of the main components of the VF ECM^3^ (Figure 5B). *Epyc* + MS1, *Mgp* + MS2, and *Postn* + /*Pi16* + MS3 determined the heterogeneity within VF fibroblasts.^10^, ^36^, ^37^ The MS1 also highly expressed *Tagln*, a smooth muscle cell marker, and the neurodevelopment‐related *Map1b*,^38^, ^39^ and this was in accordance with the GSVA results showing that MS1 is mainly involved in angiogenesis and neuronal differentiation (Figure S1). MS2 can be further divided into MS2‐1 and MS2‐2. We found several inflammatory and metabolism‐related genes enriched in MS2‐1, including *Lcn2*, *Crispld2* and *Cyp1b1*.^40^, ^41^, ^42^ MS2‐2, based on GSVA analysis, has more enrichment pathways, especially for antigen binding, assembly, and presentation (Figure 5C; Figure S1), which is in accordance with the highly expressed *Lbp* and *Tf* in MS2‐2.^43^, ^44^ We observed a unique ‘growth ring’‐like arrangement of MS1‐3 in the mesenchymal layer at the ends of the VF in which MS1 cells were located centrally with MS3 cells in the middle ring and MS2 cells in the outermost layer (Figure 5D,E). Also, the MS2 cells showed the typical long spindle shape of fibroblasts compared to the MS1 and the MS3 cells (Figure 5D1,D2,E,E1). The *Abcc9* + /*Apcdd1* + MS4 cells were identified as pericytes, wrapping around the VECs (Figure 5C,F,F1,G) and together composing the vascular system.^45^, ^46^

### 2.5 Multi‐level cell–cell communication among different cell populations

**FIGURE 6:**
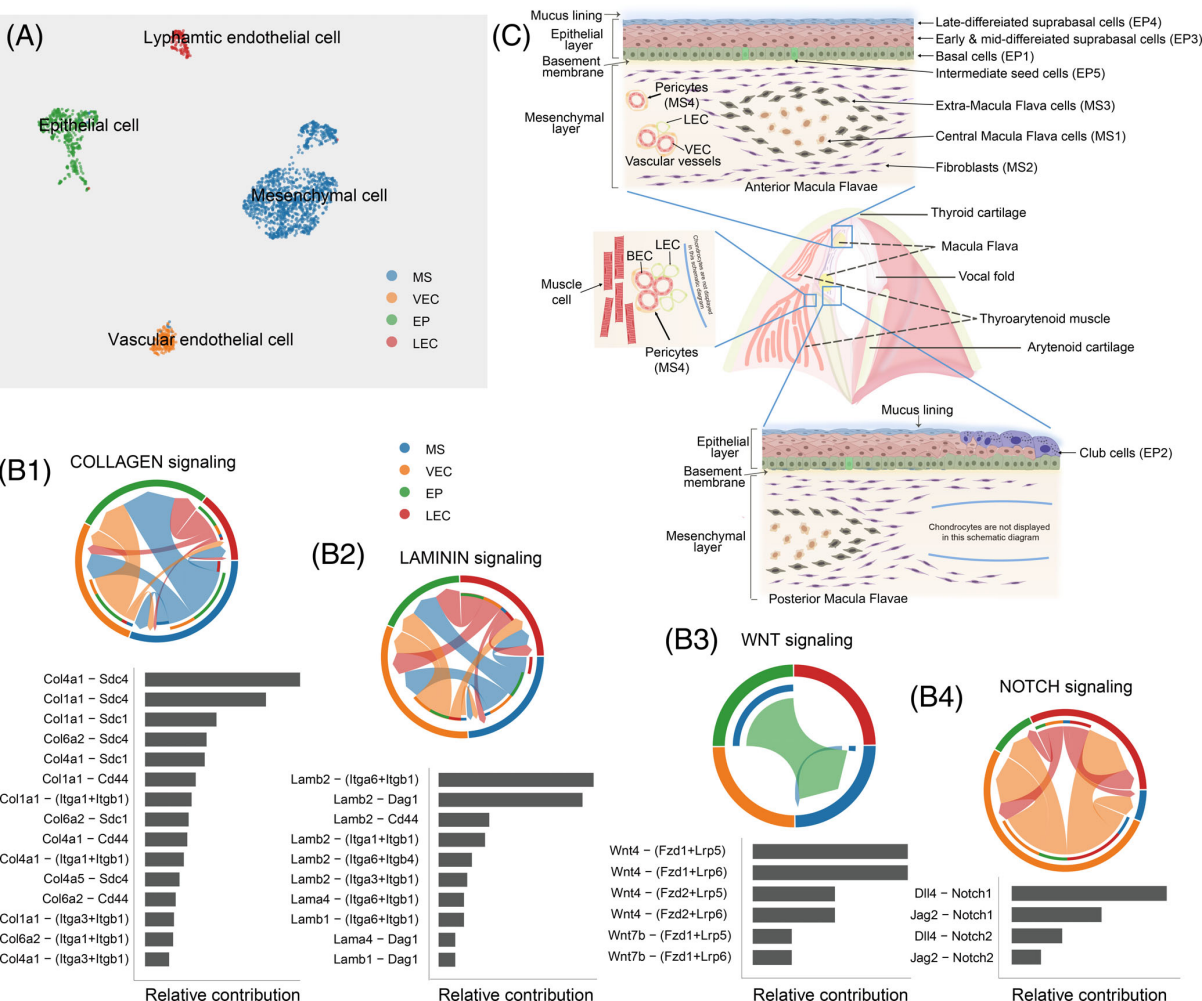
Cellular interactions and microstructure of rat vocal fold (VF). (A) Uniform Manifold Approximation and Projection for VF epithelial cells, VF mesenchymal cells (MSs) and VF endothelial cells. (B1) Chord plot for Collagen signalling with the relative contribution of its ligand‐receiver pairs below. (B2) Chord plot for Laminin signalling with the relative contribution of its ligand‐receiver pairs below. (B3) Chord plot for WNT signalling with the relative contribution of its ligand‐receiver pairs below. (B4) Chord plot for NOTCH signalling with the relative contribution of its ligand‐receiver pairs below. (C) Diagram of VF structure, including the anatomy of rat VFs (coronal position), and a schematic of the anterior commissure, anterior Macula Flava, posterior Macula Flava and the interstitial space between the thyroarytenoid muscle and the arytenoid cartilage. (C) Diagram of VF structure, including the anatomy of rat VFs (coronal position) and a schematic of cell arrangement in the anterior commissure, anterior Macula Flava, posterior Macula Flava and the interstitial space between the thyroarytenoid muscle and the arytenoid cartilage.

We next used NicheNet and CellChat for cell‐communication network analysis among structural cells (Figure 6A). We identified multiple signalling pathways within the main cell populations, including WNT, PDGF, BMP, IGF, FGF, PTN, NOTCH and non‐canonical WNT. Four signalling pathways—WNT, NOTCH, Laminin and Collagen—were further studied in detail to identify the major signalling inputs and outputs, ligand–receptor interactions, and signalling patterns. Collagen and laminin, which are two multifunctional glycoproteins that play critical roles in cellular morphogenesis, cell signalling, tissue repair, and cell migration,^47^ showed complicated networking structures with more ligand–receptor pairs. Both autocrine and paracrine patterns were detected in laminin and collagen signalling, sourced mainly from the MSs and VECs towards the EPs and VECs (Figure 6B1,B2). The EPs were the most prominent source for WNT signalling in MSs in a paracrine manner according to string plot and network centrality analysis (Figure 6B3, Figures S3 and S4). Notch signalling is a major pathway for interactions between VECs and LECs and mainly involves VECs sending signals toward LECs (Figure 6B4).

## 3 DISCUSSION

The VF is an amazingly versatile organ with complex motor functions that allow for complicated sound production. Based on the collection and analysis of 5836 high‐quality cell transcriptomes, we have created a comprehensive molecular cell atlas of healthy VF tissue from rodents. We identified five major cell groups, including EPs, MSs, ECs, immune cells and muscle cells, as well as 11 transcriptionally distinct cellular subpopulations apart from muscle cells and immune cells (Figure 6C). We revealed the cellular heterogeneity and distribution, cellular functions, and cell–cell communication patterns in structural cell types. We also discovered a novel EP type that can be critically helpful for research on the EMT process in HNSCC. Compared with the snRNA‐seq data on healthy human VF,^47^ we found high similarity in cell clustering and type‐specific marker expression in both rat and human VF tissues, despite the relatively simple structure and function of rodent VFs.

The classification of squamous EP subclusters and the club cells in the VF demonstrated the anatomic features of VF as a common pathway between the digestive and respiratory tracts.^4^ The basal cells (EP1), suprabasal cells (EP3) and superficial layer cells (EP4) constituted a continuous, gradually differentiated squamous epithelia (Figure 2A) accompanied by alterations in type‐specific markers and an increasing variety of cellular functions, indicating the increasing heterogeneity in EP subpopulations with differentiation. Meanwhile, the squamous cells and the club cells were heterogeneous, both morphologically and genetically. Also, compared to the gene expression features in the healthy human VF EPs, we compared similarities in cellular type specificity markers, such as the expression of *Krt5* and *Krt13* in differentiated cells and the expression of *Scgb1a1* and *Scgb3a1* and mucin expression (*Muc1* and *Muc5b*) in secretary cells,^47^ suggesting the conservation in VF development among species. Moreover, we also found expression of both *Muc4* and *Muc5ac* in human nasal EPs and human VF EPs, thus reflecting the tissue features along the airway.^48^

The EMT process has long been believed to be a binary process in which the epithelium transforms directly into mesenchymal cells with the loss of expression of epithelial markers and the gain of the expression of mesenchymal markers.^49^ However, our discovery of EP5 may lead to a completely new view on the process of EMT. The distinct transcriptomic characters of EP5 (*Krt15* and *Col3a1*) make it a strong candidate for further research on EMT in HNSCC tumorigenesis. EP5 cells are rare and scattered among the EP1 subpopulation in the healthy squamous epithelia, whereas a significant increase in the number of EMT cells has been observed in various malignant tissues with diverse mesenchymal‐like morphological and genetic alterations, accompanied by the loss of differentiation and the onset of malignancy. We observed high levels of EMT in the parenchyma, and thus the state of EMT cells in correlation to EP5 cell trans‐differentiation needs further investigation. The heterogeneity of the EMT cells during tumorigenesis reflected the complexity of the tumour microenvironment, implying a unique differentiation pattern that is required for tumour proliferation. Our results verified a controversial point of view from recent studies that EMT is a gradual process with several cellular states exhibiting a transcriptional and morphological intermediate state between epithelial and mesenchymal cells^32^ and added the tissue‐specific EMT markers for HNSCC. Our exciting results provide new insights into research on EMT process, and the promising EP5s show great potential in future research for HNSCC.

The ECs and MSs are important cellular components of the VF stroma. We found fewer VEC and LEC subtypes, with less different transcriptome characteristics compared to ECs from other vessel‐rich tissues such as the brain, spleen, and liver.^9^ The VECs and the MS4 pericytes form small blood vessels in the rat VF tissue, in the anterior commissure, and deep in the muscle layer or between the cartilage and the muscles, along with lymphatic vessels formed by LECs.

The fibroblast subtypes are arranged similar to ‘growth rings’, which have been identified as the Macula Flava at both ends of the VF.^50^ We accordingly identified the MS1 cluster as the central Macula Flava cells, the MS3 cluster as the extra‐Macula Flava cells, and the MS2 cluster as the fibroblasts based on transcriptomic features and spatial mapping information. However, the transcriptomic evidence in our present work suggested the lack of stemness in the MS1 and MS3 clusters. High heterogeneity was detected within fibroblasts indicating the complexity of ECM composition.

Despite the lack of further evidence for the biological function of MS1–3, we propose that the diversity of MS subpopulations may be associated with the maintenance of the VF's layered mesenchymal structures, which is commonly known as the layered structure of the lamina propria.^3^ However, the specific distribution of MS subpopulations and the microenvironment in which they exist, as well as how they perform their specific biological functions, still need to be further investigated. In addition, tissue‐specific gene expressions such as *Col3a1* and *Penk* were detected in VF MSs, suggesting a specific ECM environment fit for organ functions.^10^

Immune cells and muscle cells are also critical stromal cellular components of the VF mucosa. Nonetheless, the detailed discussion on these two cells was not developed in this work.

Of note, the loss of cellular spatial information is inherent in the scRNA‐seq technique. Also, cell signalling transduction occurs only within a limited distance. Spatial information should be further incorporated by introducing spatial transcriptomics and in situ sequencing technologies for further analysis of VF tissues.

In summary, we constructed a comprehensive transcriptome atlas of healthy rat VFs at single‐cell RNA resolution. We updated the cell composition and distribution, analysed cellular heterogeneity, and identified tissue‐specific biomarkers for subpopulation classification. Most surprisingly, we discovered a novel intermediate cell type, which can be a potential key factor for laryngeal carcinogenesis. The novel insights we present here and future discoveries arising from the utilization of our high‐resolution VF atlas might provide the basis for unveiling the physio‐pathological mechanisms at work in the VF and for promoting the development of regenerative medicine, biomaterials and novel therapeutics for malignancy.

## 4 EXPERIMENTAL MODEL AND SUBJECT DETAILS

### 4.1 Sprague–Dawley rats

The SPF Sprague–Dawley rats were purchased from qualified animal experiment centres. The rats were housed in ventilated cages in a temperature and light‐regulated room in an SPF facility and received irradiated food and sterilized water ad libitum. The rodent experiments were approved by the Institutional Animal Care and Use Committees of the Eye, Ear, Nose and Throat Hospital of Fudan University in Shanghai, China and were in compliance with all relevant ethical regulations (2019079). Male rats of around 250 g were used in the study. Larynxes were dissected after euthanasia and were fixed in 4% formaldehyde. All tissues were paraffin embedded and sectioned.

### 4.2 Human tumour specimens

Patients at the Guangdong Provincial People's Hospital (GDPH) gave consent pre‐operatively to take part in the study following Institutional Review Board approval (KY‐Q‐2022‐478‐01). The characteristics of the human subjects who provided samples are summarized in Table S1. Fresh biopsies of HNSCC and PVL were collected at the time of surgical resection and were selected from the primary tumour or lesion dissection. Surgical resections were fixed in 10% buffered formalin immediately upon removal. All tissues were embedded in optimal cutting temperature compound (OCT) and sectioned.

## 5 METHOD DETAILS

### 5.1 Single cell suspension

Fresh larynxes were dissected and washed with cold phosphate‐buffered saline (PBS; Bioss, Beijing, China). VFs were immediately prepared for single‐cell separation, and most of the cartilage was removed for optimal tissue digestion in order to maintain the viability of individual cells. The VFs were dissociated with TrypLE Express Enzyme (1×) for 15 min at 37°C (Gibco, ThermoFisher Scientific) according to the manufacturer's guidelines. Viability was confirmed to be >85% in all samples using a LIVE/DEAD Viability/Cytotoxicity kit (ThermoFisher Scientific). Cell suspensions were filtered through a 70 mm filter (ThermoFisher Scientific), and cells were washed once in 1× PBS with 0.04% bovine serum albumin (BSA; Sigma‐Aldrich, Shanghai, China) and resuspended in 1× PBS with 0.04% BSA before sorting.

### 5.2 cDNA synthesis and library construction

The 10x Genomics Chromium platform was used for single‐cell sequencing. Cell suspensions were loaded into the Chromium Controller Instrument to be encapsulated into GEMs (gel beads in emulsion). The Chromium Next GEM Single Cell 3′ Library & Gel Bead Kit v3.1, the Chromium Next GEM Chip G Single Cell Kit, and the Chromium i7 Multiplex Kit were used for all downstream processes, including reverse transcription, cDNA amplification, and library construction. A total of 10,000 cells were targeted for library preparation following the pipeline from the manufacturer. The indexed sequencing libraries were run on an Illumina sequencing system for paired‐end sequencing. Raw fastq files were aligned to the rat genome (mRatBN7.2) and aggregated using Cell Ranger (v7.0.1), thus generating expression matrices based on quantified data.

#### 5.2.1 Immunofluorescence

Immunofluorescence was performed as previously described by You et al.^51^. Briefly, permeabilization in 0.1% Triton X‐100 was selectively applied according to the position of the target proteins. This was followed by blocking with 10% normal goat serum diluted with PBS for 1 h and incubation with primary antibodies of different species overnight at 4°C. The sections were washed three times with PBS followed by Alexa Fluor‐conjugated secondary antibodies for 1–2 h at room temperature on the following day. The Alexa Fluor‐conjugated primary antibodies were incubated when needed after completion of the previous staining and re‐blocking. The sections were mounted with mounting media with DAPI. All procedures were performed according to the manufacturers' instructions. Fluorescence images were acquired using a ZEISS LSM 900+ Airyscan2 confocal microscope, and quantification was performed using ImageJ software.

### 5.3 Statistical analysis

Statistical analyses were performed with RStudio V1.4 (RStudio IDE, Boston, MA) or GraphPad Prism version 9 (GraphPad Software, La Jolla, CA). Parameters such as sample size, the number of replicates, the number of independent experiments and measures of centre, dispersion and precision (mean ± SEM) are reported in the figures and figure legends.

### 5.4 scRNA‐seq data clustering and dimensionality reduction

The Seurat R package (v4.1.1) was used for the analysis of gene expression levels and cell clustering. Counts were first normalized using SCTransform (v0.3.3). Low‐quality cells were filtered out based on reads mapped to each cell and the mitochondrial percentage (nFeature_RNA <7500, nFeature_RNA >200, percent.mt <15). Dimensionality reduction and cell clustering were performed using principal component analysis (RunPCA) on highly variable genes and UMAP dimension reduction (RunUMAP). Clusters were identified with the FindNeighbors and FindClusters functions. Marker genes of the defined cluster were visualized using the DotPlot function. Cell types with functional identities were assigned to cell clusters based on previously reported results. For immune cell type identification, we referred to the automatic annotation method SingleR (v1.10.0) based on the built‐in reference index celldex (v1.6.0).

### 5.5 Cell–cell communication analysis and network visualization

The R toolkit CellChat (v1.5.0) was used to evaluate cellular communication across different cell types through quantitative characterization and network analysis of intercellular communication from normalized expression profiles in the scRNA‐seq data. We prepared the ligand and receptor information for analysis of rat data based on the information in CellChatDB. Homologous genes in these files were mapped to rats using the Ensembl (mRatBN7) biomaRt (v2.52.0) getLDS function. The cell–cell communication network of each signalling pathway was visualized using chord diagrams. Ligand–receptor pairs making significant contributions to the overall signalling were listed. Various incoming and outgoing signalling proportions in a particular cell group were visualized using a heatmap, which simultaneously illustrated the dominant sending and receiving cell group of each signalling pathway. We also performed ligand–target network prediction using the nichenetr (v1.1.0) R package following the same homologous gene mapping process as mentioned above. Circos plot was used for visualizing active ligand‐target links between interacting cells.

### 5.6 Gene set variation analysis (GSVA)

We used the GSVA package (v1.44.2) in R to perform GSVA based on cell cluster average gene expression matrices. Pathway analyses were predominantly performed on gene sets derived from Gene Ontology Biological Process (GO‐BP) exported using the MSigDB database (v 7.5.2) and finally presented after removal of duplicates and threshold filtering.

#### 5.6.1 Cell counting process

ImageJ with the Cell Counter plugin was used for automatic cell counting. RGB images were analysed directly by Selecting Plugins → 1 analysis → Cell Counter. The cell number was automatically counted when the colour and the object were properly selected. The resulting log file was exported as an Excel spreadsheet. The reagents and software used in this work have been listed in Table S2.

## AUTHOR CONTRIBUTIONS

Danling Liu: Methodology; writing—original draft preparation; funding acquisition. Yunzhong Zhang and Luo Guo: Data curation. Wen Li, Jin Guo, Peifang Li, Tingting Qian, Liping Zhao and Xiaoning Luo: Methodology; investigation and software. Shan Sun, Jun Shao, Siyi Zhang and Rui Fang: Conceptualization; visualization; supervision; writing—reviewing and editing; funding acquisition and project administration.

## CONFLICT OF INTEREST STATEMENT

The authors declare no conflicts of interest.

## Supporting information


**Data S1.** Supporting information.

Table S1.

Table S2.

## Data Availability

The sc‐RNA seq raw data will be made available on request toward the Lead Contact, Shan Sun (Shansun@fudan.edu.cn).
