# Supplementary material for: Single‐cell atlas of healthy vocal folds and cellular function in the endothelial‐to‐mesenchymal transition
Source: Cell Prolif. 2024 Sep 8;57(12):e13723. doi: 10.1111/cpr.13723 (PMC11628749; doi:10.1111/cpr.13723)
Supplement: Supplementary file 1 — Data S1. Supporting information. [file CPR-57-e13723-s001.docx]

**Supplementary Data**


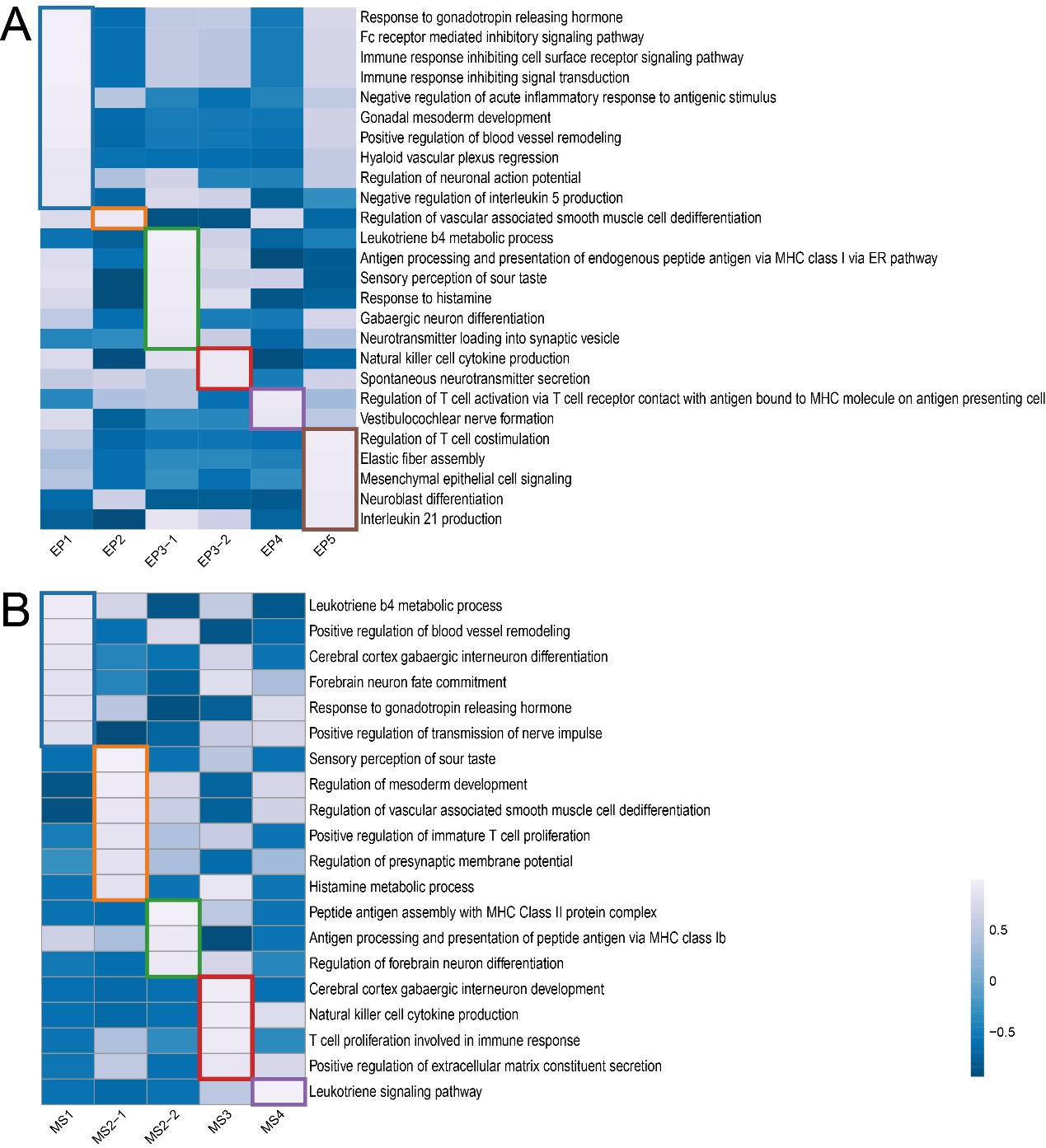


**Fig. S1 Heatmap of functional enrichment.** A. cellular functions of EPs; B. Cellular functions of MSs. The color indicates the average expression level; the lighter the color, the stronger the expression.


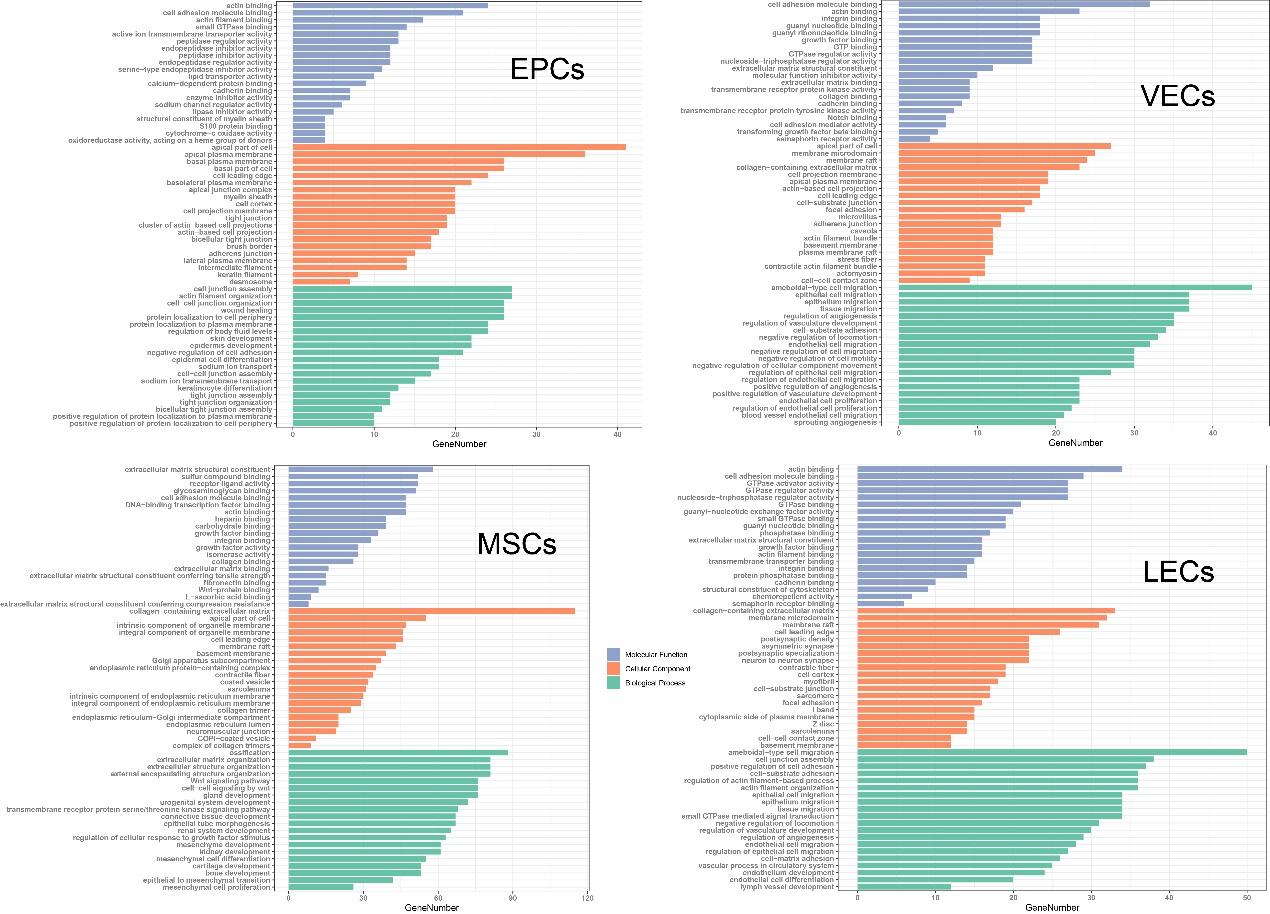


**Fig. S2 Gene ontology (GO) analysis of major structural cells.** GO data presented the enriched functions of molecular function (blue), biological process (orange), cellular component (green) for major structural cells. EPCs = epithelial cells, MSCs=mesenchymal cells, VECs = vascular endothelial cells, LECs = lymphatic endothelial cells.


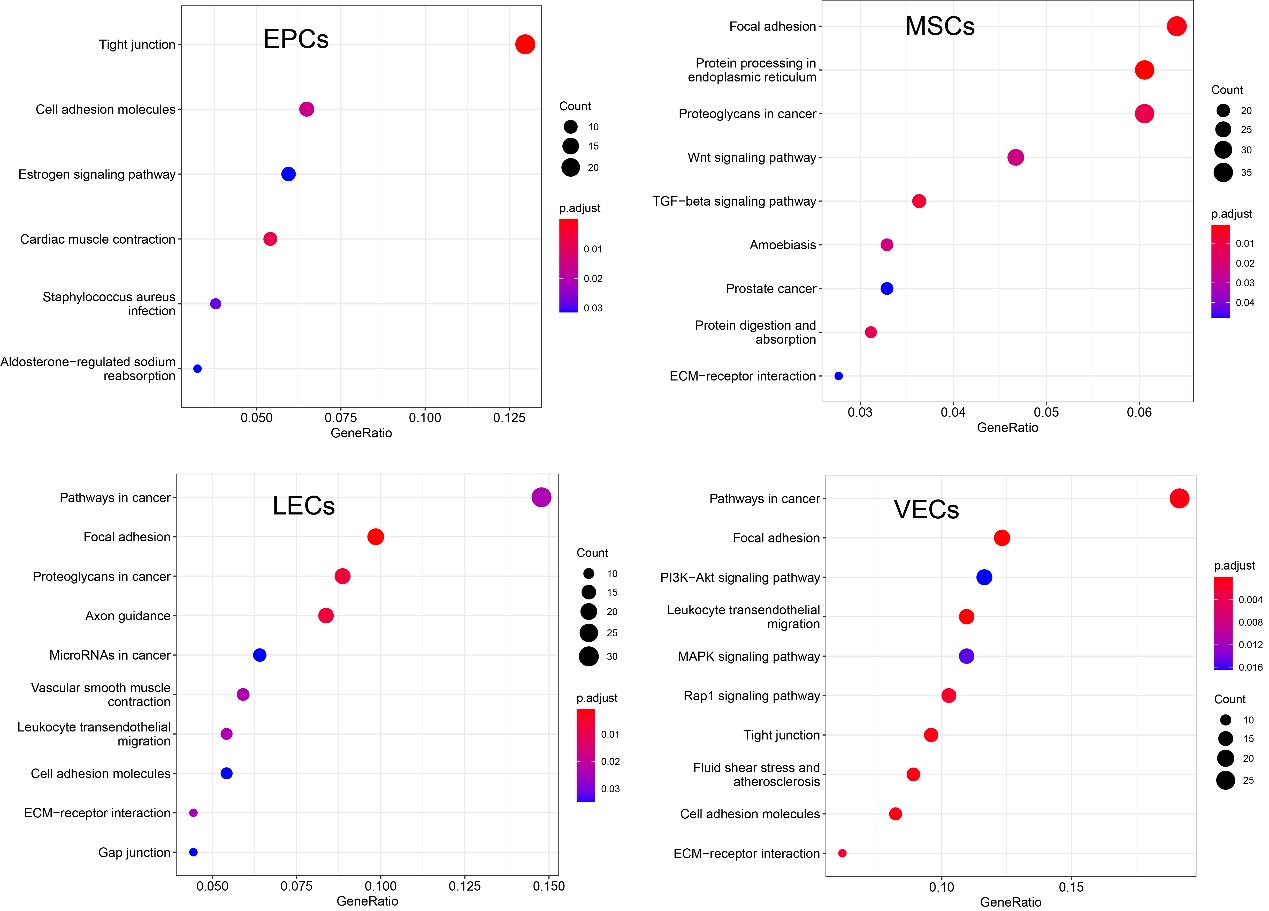


**Fig. S3 KEGG of major structural cells.** KEGG data present the enriched signaling pathways for major structural cells. EPCs = epithelial cells, MSCs = mesenchymal cells, VECs = vascular endothelial cells, LECs = lymphatic endothelial cells. The size of the dot indicates the percentage of cells within a cell type, and the red color represents a high expression level and the blue color represents a low expression level.


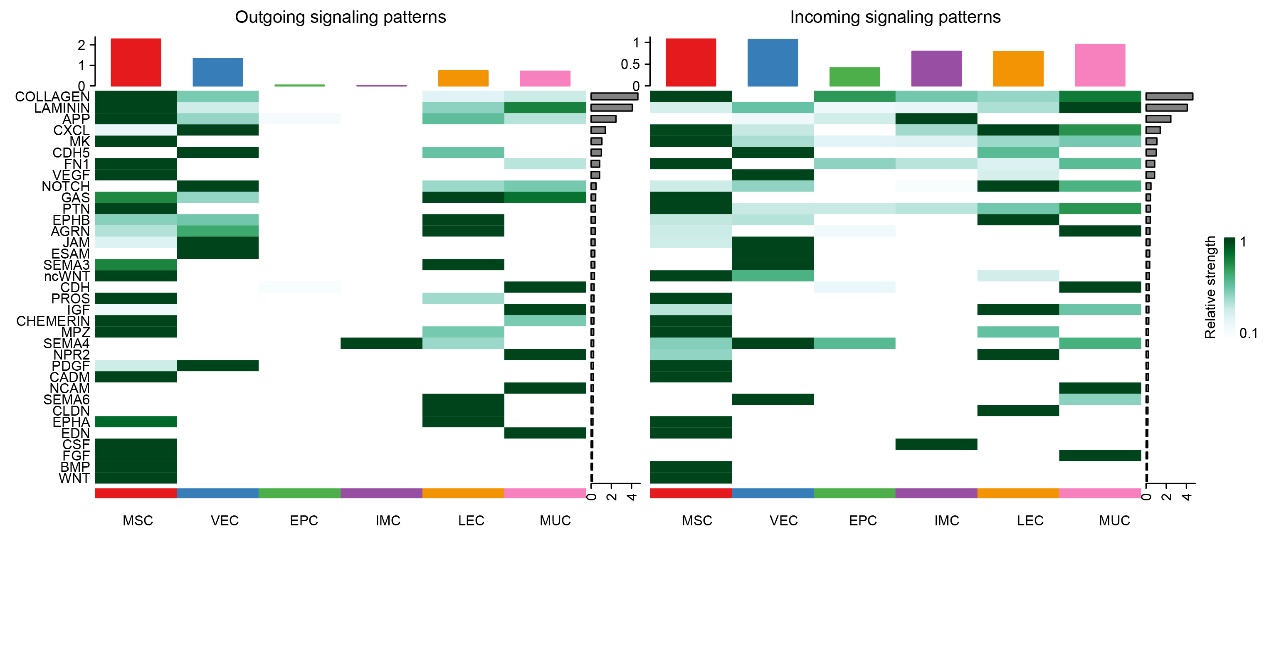


**Fig. S4: Outgoing and incoming signaling patterns of five major VF cell types.** The color indicates the average expression level; the darker the color, the stronger the expression.
