# Supplementary material for: Single‐cell atlas of healthy vocal folds and cellular function in the endothelial‐to‐mesenchymal transition
Source: Cell Prolif. 2024 Sep 8;57(12):e13723. doi: 10.1111/cpr.13723 (PMC11628749; doi:10.1111/cpr.13723)
Supplement: Supplementary file 2 — Table S1. [file CPR-57-e13723-s003.docx]

**Table S1. Patients included in the experiment**

| Patient ID | Age/Sex | Primary Site | Lymph Nodes  Collected | Pathologic  Stage | Grade | PNI | LVI | ECE |
| --- | --- | --- | --- | --- | --- | --- | --- | --- |
| 978644 | 60/M | Left VF | Left & Right level II-IV | T4AN0M0 | 4 | Present | Absent | Present |
| 2096069 | 79/M | Left VF | _ | T1AN0M0 | 1 | Absent | Absent | Absent |
| 2069072 | 61/M | PVL in Left VF | _ | / | / | / | / | / |

PNI = perineural invasion; LVI = lymphovascular invasion; ECE = extracapsular extension;

PVL = proliferative verrucous leukoplakia; VF = vocal fold
