# Supplementary material for: Single‐cell atlas of healthy vocal folds and cellular function in the endothelial‐to‐mesenchymal transition
Source: Cell Prolif. 2024 Sep 8;57(12):e13723. doi: 10.1111/cpr.13723 (PMC11628749; doi:10.1111/cpr.13723)
Supplement: Supplementary file 3 — Table S2. [file CPR-57-e13723-s002.docx]

**Table S2. Reagents and Resources**

| REAGENT or RESOURCE | SOURCE | IDENTIFIER |
| --- | --- | --- |
| Antibodies | | |
| Mouse monoclonal Anti-Cytokeratin 15 antibody [LHK15] | Abcam | Cat#ab80522  RRID:AB_1603675 |
| Recombinant Rabbit monoclonal Anti-FABP4 antibody [EPR3579] | Abcam | Cat#ab92501  RRID:AB_10562486 |
| Mouse monoclonal Reelin Antibody (G10) - BSA Free | Novus Biologicals | Cat# NB600-1081  RRID:AB_2179306 |
| Rabbit polyclonal Anti-Caveolin-1 antibody | Abcam | Cat#ab2910  RRID:AB_303405 |
| Armenian hamster Monoclonal MUC1 Antibody (MH1 (CT2)) | Invitrogen | Cat# MA5-11202  RRID:AB_2897740 |
| Rabbit polyclonal MUC20 Polyclonal Antibody | Invitrogen | Cat# PA5-75164  RRID:AB_2718892 |
| Mouse monoclonal Mucin 5B/MUC5B Antibody (5B#19-2E) | Santa cruz | Cat# sc-21768  RRID:AB_627975 |
| Cytokeratin 13 Recombinant Rabbit Monoclonal Antibody (ARC1824) | Invitrogen | Cat# MA5-37816 RRID:AB_2837588 |
| Rabbit monoclonal Anti-beta 2 Microglobulin [EP2978Y] Antibody | Abcam | Cat# ab75853, RRID:AB_1523204 |
| Rabbit polyclonal POSTN Antibody | Affinity Biosciences | Cat# DF6746, RRID:AB_2838708 |
| Rabbit Anti-Collagen III Polyclonal Antibody, Cy3 Conjugated | Bioss | Cat# bs-0948R-Cy3, RRID:AB_11112738 |
| IHC‑plus™ Monoclonal Mouse anti‑Bovine MAP1B Antibody | LSBio | Cat# LS-B5165-50, RRID:AB_10851664 |
| Recombinant Alexa Fluor® 488 Anti-Cytokeratin 17 antibody [EP1623] - Cytoskeleton Marker | Abcam | Cat# ab185032, RRID:AB_2889195 |
| Rabbit Anti-Uteroglobin Polyclonal Antibody, Cy3 Conjugated | Bioss | Cat# bs-1935R-Cy3, RRID:AB_11088025 |
| IHC‑plus™ Polyclonal Rabbit anti‑Human MGP / Matrix Gla‑Protein Antibody | LSBio | Cat# LS-B14824 |
| Rabbit Anti-LMO4 Polyclonal Antibody, Cy3 Conjugated | Bioss | Cat# bs-5966R-Cy3, RRID:AB_11098535 |
| Mouse monoclonal ABCC9 Antibody (S319A-14) | Novus Biologicals | Cat# NBP2-22403 |
| Goat Anti-Mouse IgG H&L (Alexa Fluor® 488) | Abcam | Cat# ab150113, RRID:AB_2576208 |
| Goat Anti-Mouse IgG H&L (Alexa Fluor® 647) | Abcam | Cat# ab150115, RRID:AB_2687948 |
| Goat Anti-Rabbit IgG H&L (Alexa Fluor® 555) | Abcam | Cat# ab150078, RRID:AB_2722519 |
| Donkey Anti-Rabbit IgG H&L (Alexa Fluor® 647) | Abcam | Cat# ab150078, RRID:AB_2722519 |
| Goat Anti-Rabbit IgG H&L (Alexa Fluor® 488) preadsorbed | Abcam | Cat# ab150081, RRID:AB_2734747 |
| Goat Anti-Armenian Hamster IgG H&L (Alexa Fluor® 568) | Abcam | Cat# ab175716, RRID:AB_2924663 |
| Bacterial and virus strains | | |
| No use |  |  |
| Biological samples |  |  |
| See Table S1 for a list of patients included in the study. |  |  |
| Chemicals, peptides, and recombinant proteins | | |
| No use |  |  |
| Critical commercial assays | | |
| No use |  |  |
| Deposited data | | |
| Raw data |  |  |
| Experimental models: Cell lines | | |
| No use |  |  |
| Experimental models: Organisms/strains | | |
| No use |  |  |
| Oligonucleotides | | |
| No use |  |  |
| Recombinant DNA | | |
| No use |  |  |
| Software and algorithms | | |
| GraphPad Prism version 9.0 | GraphPad Software | https://www.graphpad.com/scientific-software/prism/ |
| R studio 1.4 | RStudio IDE | https://www.rstudio.com/categories/rstudio-ide/ |
| Image J | National Institutes of Health | https://imagej.nih.gov/ij/ |
| ZEISS ZEN 3.2 (blue edition) | ZEISS | https://www.zeiss.com.cn/microscopy/products/microscope-software/zen.html#inpagetabs-5 |
| Other | | |
| No use |  |  |
